# Synthesis, Crystal Structure and Biological Activity of 2-Hydroxyethylammonium Salt of *p*-Aminobenzoic Acid

**DOI:** 10.1371/journal.pone.0101892

**Published:** 2014-07-23

**Authors:** Manuela E. Crisan, Paulina Bourosh, Massimo E. Maffei, Alessandra Forni, Stefano Pieraccini, Maurizio Sironi, Yurii M. Chumakov

**Affiliations:** 1 Department of Organic Chemistry, Institute of Chemistry Timisoara of Romanian Academy, Timisoara, Romania; 2 Laboratory of Physical Methods of Solid State Investigation “T. Malinowski”, Institute of Applied Physics, Academy of Sciences of Moldova, Chisinau, Republic of Moldova; 3 Department of Life Sciences and Systems Biology, Plant Physiology Unit, Innovation Centre, University of Turin, Torino, Italy; 4 ISTM-CNR, Institute of Molecular Sciences and Technologies of CNR and INSTM UdR, Milano, Italy; 5 Department of Chemistry and INSTM UdR, University of Milan, Milano, Italy; University of Akron, United States of America

## Abstract

*p*-Aminobenzoic acid (*p*ABA) plays important roles in a wide variety of metabolic processes. Herein we report the synthesis, theoretical calculations, crystallographic investigation, and *in vitro* determination of the biological activity and phytotoxicity of the *p*ABA salt, 2-hydroxyethylammonium *p*-aminobenzoate (HEA-*p*ABA). The ability of neutral and anionic forms of *p*ABA to interact with TIR1 pocket was investigated by calculation of molecular electrostatic potential maps on the accessible surface area, docking experiments, Molecular Dynamics and Quantum Mechanics/Molecular Mechanics calculations. The docking study of the folate precursor *p*ABA, its anionic form and natural auxin (indole-3-acetic acid, IAA) with the auxin receptor TIR1 revealed a similar binding mode in the active site. The phytotoxic evaluation of HEA-*p*ABA, *p*ABA and 2-hydroxyethylamine (HEA) was performed on the model plant *Arabidopsis thaliana* ecotype Col 0 at five different concentrations. HEA-*p*ABA and *p*ABA acted as potential auxin-like regulators of root development in *Arabidopsis thaliana* (0.1 and 0.2 mM) and displayed an agravitropic root response at high concentration (2 mM). This study suggests that HEA-*p*ABA and *p*ABA might be considered as potential new regulators of plant growth.

## Introduction

The interest in carboxylate compounds in general and benzoic acid derivatives in particular continues to grow because of their chemical and biological properties. Benzoic acids and their derivatives are important structural elements for many natural products, being involved in various physiological processes in plants. Among these compounds, *p*-aminobenzoic acid (*p*ABA) has displayed biological, medicinal and industrial interest. *p*ABA is a compound of high biological significance, it is present in plant and animal tissues and is sometimes referred to as bacterial vitamin H_1_, B_x_, or B_10_ [1]. *p*ABA is a well-known precursor of folic acid [2], and has recently been identified as a coenzyme Q precursor [3], [4], which opens new possibilities for its application in medicine. It is a building block used in design of drugs and frequently found as a structure moiety in drugs [5]. *p*ABA exhibits a wide range of therapeutic uses as antioxidant [6], [7], antibacterial [8], [9], antimutagenic [10], anticoagulant [11], [12], fibrinolytic and immunomodulating agent [13], protective drug against UV-irradiation [14]–[16] and in diagnostic tests for the state of the gastrointestinal tract [17], [18].

Recent studies in agriculture report the role of *p*ABA in plant thermotolerance [19] and its action as chemical inducer of systemic acquired resistance against plant pathogens [20]. Considered to be in the B-complex vitamin family and characterized by safety and cost effectiveness, *p*ABA is frequently added along with plant hormones in nutriment media as a stimulator of seed germination [21]. To the best of our knowledge, the role of folate precursor *p*ABA as a plant growth modulator has not been investigated so far.

Besides the plethora of properties noted above, *p*ABA is a versatile reagent for structure extension through linear hydrogen bonding associations, through both the carboxylic and amine functional groups [22]. Keeping in view the structural and biological diversity of *p*ABA and in connection with our interest in supramolecular chemistry of carboxylate salts [23], [24], here we report the synthesis, theoretical calculations, crystallographic investigation, and *in vitro* phytotoxic activity of 2-hydroxyethylammonium *p*-aminobenzoate (HEA-*p*ABA). As a base, we chose the natural amino alcohol 2-hydroxyethylamine (HEA), a suitable model of alkanolamine. This compound is cheap, commercially available, environmentally tolerable and has hydrogen-bond donor sites. On the other hand, HEA is an essential component of cell membranes and an important metabolite in plants for the synthesis of choline and membrane lipids, such as phosphatidylethanolamine and phosphatidylcholine [25].

The preparation of multicomponent organic crystals (solvates, hydrates, co-crystals, and salts) from such multifunctional molecules as benzoic acids and alkanolamine display interesting networks and a wide range of different biological properties [23], [24], [26]–[28]. The possible binding modes of *p*ABA, its anionic form and the natural auxin IAA bound to the auxin receptor TIR1 have been evaluated by docking experiments and refined by Molecular Dynamics (MD) and Quantum Mechanics/Molecular Mechanics (QM/MM) calculations. The present work continues our recent investigations of new structural aspects of alkanolamine salts [24], reports the full details of the X-ray crystallographic structure of HEA-*p*ABA and introduces this compound and corresponding acid as new potential regulators of plant growth.

## Materials and Methods

### General

*p*ABA and HEA used for the synthesis of HEA-*p*ABA were of analytical grade and purchased from Fluka AG (Buchs SG). HEA was freshly distilled before any use. FT-IR spectra in the range 4000−400 cm^−1^ were recorded with a JASCO − FT/IR-4200 spectrometer, using the KBr pellet technique with a resolution of 4.0 cm^−1^ and a scanning speed of 16 mm s^−1^. The optical properties of HEA-*p*ABA were examined by using a UV−Vis spectrophotometer at room temperature. UV−visible spectra were recorded in the 190−800 nm range with PERKIN-ELMER LAMBDA 12 UV−Vis spectrometer. Melting point was found for finely purifying compound (by repeated recrystallization) using a Boetius instrument.

### Synthesis and characterization of HEA-pABA

HEA-*p*ABA was prepared by the reaction of HEA and *p*ABA in a 1∶1 molar ratio. Under vigorous stirring, freshly distilled HEA was added drop wise to the solution of the acid in acetone, at reflux for 2 h to complete the reaction. The formed salt was precipitated in a white crystalline state and, after cooling at room temperature, collected by filtration, washed with cold acetone, and dried under vacuum for 3 h. This reaction had a 90% yield. The salt was recrystallized to obtain suitable crystals for X-ray analysis. Crystals were grown by slow evaporation of ethanol solution at room temperature. The purity of synthesized salts was 99.3% (±0.8%), established by spectrophotometric method. This consisted in determining the specific extinction (absorbance at λ_max_ of a solution 1 gl^−1^, in a cell of 1 cm width) of the free acid (ε_a_) and the corresponding salt (ε_s_), for the UV maxima: 
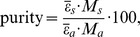
where *M_s_*, and *M_a_* are the molecular weights for the salt and free acid, respectively. The elemental analysis was in agreement with the expected stoichiometry. The FT-IR spectrum (KBr pellets prepared by grinding a 5−10 mg sample with 100 mg KBr) was consistent with salt formation.

C_9_H_14_N_2_O_3_, (198.22), m.p. 147–150°C; λ_max_ = 265.19 nm; FT-IR spectra (KBr pelet, cm^−1^): 3392, 3227, 2132, 1591, 1540, 1384, 1361, 1073, 1016; calcd. (%): C 54.53, H 7.12, N 14.13; found (%): C 54.51, H 7.03, N 14.10.

### X-ray measurement and refinement

X-ray data for HEA-*p*ABA were collected at room temperature on a Siemens P3/PC diffractometer (CuK_α_-radiation). The crystallographic data and the experimental details are summarized in Table 1. Structure solution and refinement were carried out using the SHELX-97 program [29]. Non-hydrogen atoms were refined anisotropically. The hydrogen atoms were placed in calculated positions with their isotropic displacement parameters riding on those of parent atoms and the H-atoms of NH_2_ group of HEA-*p*ABA were found from differential Fourier maps. The geometric parameters for H–bonds are listed in Table 2. The geometric parameters were calculated and the figures were drawn with the use of the PLATON program [30]. Bond lengths (Å) and angles (°) for HEA-*p*ABA are listed in Table 3. The hydrogen atoms that are not involved in the hydrogen bonding were omitted from the representation of the crystal packing. Crystallographic data for HEA-*p*ABA were deposited in the Cambridge Crystallographic Data Center (CCDC 936586).

**Table 1:** Crystal Data and Structure Refinement for HEA-*p*ABA.

Compound	HEA-*p*ABA
Empirical formula	*C_9_H_14_N_2_O_3_*
Formula weight	198.22
Wavelength	1.54184
Crystal system	Orthorhombic
Space group	*Pbca*
*a*, Å	11.898(2)
*b*, Å	8.3140(17)
*c*, Å	20.790(4)
α, deg	90
β, deg	90
γ, deg	90
*V*, Å^3^	2056.5(7)
*Z*	8
Density (calc.), mg/m^3^	1.280
Absorption coefficient, mm-1	0.808
*F*(000)	848
Crystal size, mm3	0.14×0.10×0.08
θ range for data collection, deg	4.25–70.06
Index ranges	−14≤*h*≤14, −10≤*k*≤1, −25≤*l*≤24
Reflections collected/unique	1952/1952, *R*(int) = 0.0000
Completeness (%)	99.9 (θ = 70.06)
Data/parameters	1952/135
GOF on *F*2	0.998
Final *R* indices (*I*>2σ (*I*))	*R*1 = 0.0467, *wR*2 = 0.0930
*R* indices (all data)	*R*1 = 0.1101, *wR*2 = 0.1105
Largest diff. peak and hole, e.Å^−3^	0.196/−0.193

**Table 2:** Hydrogen-bonding geometry (Å) for compound HEA-*p*ABA.

No. of HB	D−H^…^A	d(D^…^H), Å	d(H^…^A), Å	d(D A), Å	∠(DHA), deg.	Symmetry transformation for H-acceptor
1	N(1)–H^…^O(3)	0.89	1.91	2.801(3)	175	−*x*+2, −*y*, −*z*+2
2	N(1)–H^…^N(2)	0.89	2.22	3.051(4)	155	*x*, −*y*+1/2, *z*+1/2
3	N(1)–H^…^O(2)	0.89	1.87	2.752(3)	171	*x*, *y*, *z*
4	O(1)–H^…^O(3)	0.82	1.90	2.715(3)	176	*x*, *y*, *z*
5	N(2)–H^…^O(1)	0.91(3)	2.29(3)	3.087(3)	146	−*x*+2, *y*−1/2, −*z*+1/2
6	N(2)–H^…^O(3)	0.82(3)	2.20(3)	3.025(3)	174	*x*+1/2, *y*, −*z*+1/2
7	C(2)–H^…^O(2)	0.97	2.41	3.247(3)	145	*x*−1/2, −*y*+1/2, −*z*+1

**Table 3:** Bond lengths (Å) and angles (°) for HEA-*p*ABA.

N(1)-C(1)	1.481(3)
O(1)-C(2)	1.409(3)
C(1)-C(2)	1.495(4)
N(2)-C(6)	1.404(3)
O(2)-C(9)	1.257(3)
O(3)-C(9)	1.281(3)
C(3)-C(4)	1.384(3)
C(3)-C(8)	1.391(3)
C(3)-C(9)	1.492(3)
C(4)-C(5)	1.377(3)
C(5)-C(6)	1.387(4)
C(6)-C(7)	1.389(4)
C(7)-C(8)	1.379(3)
N(1)-C(1)-C(2)	112.1(3)
O(1)-C(2)-C(1)	113.9(3)
C(4)-C(3)-C(8)	117.8(2)
C(4)-C(3)-C(9)	121.8(2)
C(8)-C(3)-C(9)	120.3(3)
C(5)-C(4)-C(3)	121.4(3)
C(4)-C(5)-C(6)	120.7(3)
C(5)-C(6)-C(7)	118.3(2)
C(5)-C(6)-N(2)	121.5(3)
C(7)-C(6)-N(2)	120.1(3)
C(8)-C(7)-C(6)	120.7(3)
C(7)-C(8)-C(3)	121.1(3)
O(2)-C(9)-O(3)	122.9(3)
O(2)-C(9)-C(3)	118.6(3)
O(3)-C(9)-C(3)	118.5(3)

### Docking and QM/MM calculations

The natural auxin (indole-3-acetic acid, IAA) and *p*ABA, the last one both in the neutral and anionic form, were docked in the auxin binding site as identified in the crystal structure of the TIR1 protein-auxin complex retrieved from protein data bank (PDB id 2P1P) [31] using AutoDock 4.2 software [32]. This software considers only the polar hydrogen atoms during the docking process. A Lamarkian genetic algorithm [33] was employed for the docking simulation, performing 100 independent runs per molecule. In each run, a population of 50 individuals evolved along 27000 generations and a maximum number of 25 million energy evaluations were performed. The best fit (lowest docked energy) solutions of the 100 independent runs were stored for subsequent analysis.

Docking results were refined by performing molecular dynamics simulations using the AMBER 11 program suite [34], the AMBER99SB force field [35] for the protein and the GAFF force field for ligands [36]. Each of the three protein-ligand complexes was first submitted to a geometry optimization consisting of 1000 steps of steepest descent, followed by 9000 steps of conjugate gradient, energy minimization. The systems were then equilibrated for 100 ps in the NVT ensemble and for additional 100 ps in the NPT ensemble. Ten ns long molecular dynamics simulations were then run in the NPT ensemble at 300 K and 1 atm. Water solvent was modelled with the TIP4P water model [37]. A cutoff of 12 Å was used for non-bonded VdW interactions, while electrostatic interactions were treated with the Particle Mesh Ewald approach [38].

QM/MM calculations were performed on the final structures obtained from MD simulations, using the two-layer ONIOM model [39]–[41] as implemented in Gaussian09 [42]. Owing to the high complexity of the examined system, QM/MM calculations were performed *in vacuo*, including only the ligands and the residues that showed the most favorable interactions with the ligands during the MD simulations (namely Arg403, Ser438 and Leu439) in the QM layer. The protein was included in the MM layer and its geometry was kept fixed. The QM part was treated at Density Functional Theory (DFT) level with the B3LYP functional [43], [44] and the 6–31+G* basis set. Protein atom charges were assigned using the Amber Force Field [34]. The mutual electrostatic interaction between the ligand and the protein was treated using the electrostatic embedding approach [41]. In this model, the point charges of the MM layer are included in the Hamiltonian of the QM layer, introducing a polarization of the wave function.

### Biological activity

#### Plant material and seed germination

*Arabidopsis thaliana*, ecotype Columbia 0 (Col 0) seeds were surface sterilized by treatment with a 5% (w/v) calcium hypochlorite solution and 0.02% (v/v) Triton X-100 in 80% ethanol (EtOH), for 10–12 minutes at 25–28°C, by continuous shaking. They were then rinsed twice with 80% EtOH, washed with 100% EtOH and finally with sterile distilled water [45]. Thirty sterile seeds were sown on every square Petri plate (12×12 cm) contained: 80 ml of sterile solid MS agar medium (modified from [46]), HEA-*p*ABA, *p*ABA or HEA at five concentrations (0.02, 0.1, 0.2, 1 and 2 mM) and IAA at five concentrations (0.01, 0,1, 1, 10 and 100 nM). The salt and IAA were dissolved in 5 mM MES (2-[*N*′-morpholino] ethanesulfonic acid) buffer, while *p*ABA, which is slightly soluble in water, was dissolved in MES buffer – EtOH solution, with a final EtOH concentration of 0.2% (v/v). Our previous research on *Arabidopsis thaliana* (Col 0) [28] and *Cucumis sativus* L. [27] seeds show that 0.2% (v/v) ethanol concentration in culture media does not alter germination rate of seeds, but inhibit slowly primary root length and lateral root formation, as it is confirmed by some studies reported in the literature [47], [48]. For clarity, control corresponding to *p*ABA was noted CONTROL 0.2% EtOH and for HEA-*p*ABA and HEA, CONTROL. HEA-*p*ABA, *p*ABA or HEA solved in the solvents mentioned above were sterilized by filtration (Millipore) and added to molten control agar (∼50°C). Five repetitions for each compound concentration were performed. The plates were stratified horizontally in darkness at 4°C for 2 days to break dormancy and synchronize germination, and then grown in a climate chamber at 22°C in a vertical position under a photoperiod of 16 h light (220 µmol m^−2^ s^−1^) and 8 h darkness.

### Data analysis

After 5 and 10 days, respectively, images of the Petri dishes were generated by scanning the individual plate on an EPSON Perfection 4870 Photo scanner at 600 DPI. Obtained images were digitized using the ImageJ software package, and primary root length was measured for each seedling. After 10 days, plants were stained with 0.1% (w/v) safranin for 1–2 h, then rinsed with distilled water and 100, 80, 50, 30, 15% EtOH, respectively, and mounted in 50% (v/v) glycerol. All lateral roots emerging from the primary one were observed using a Nikon YS 100 microscope 10 x objective, and were taken into account for lateral root number data.

### Statistical analysis

For all experiments, the overall data were statistically analysed by SYSTAT 10.0 program. All values are expressed as a mean ± S.E. One-way ANOVA analyses, followed by *t*-test using the Bonferroni correction, were used to compare the data obtained for primary root length, lateral root number at 5 and 10 days for treatments (HEA-*p*ABA, *p*ABA and HEA) at five different concentrations and controls. Different letters are used to indicate means that differ significantly (P<0.05).

## Results and Discussion

### Synthesis and characterization of HEA-pABA

HEA-*p*ABA is a stable solid salt in accordance with the appropriate “rule of three”. The value of ΔpKa (the difference between the pKa value for HEA and *p*ABA) was estimated to be >3, that determines the extent of proton transfer. UV spectroscopic data obtained in 0.1 M NaOH showed similar λ_max_ values for both salt and acid, that confirms the existence of the same anion in both compounds (see Figure S1). The cut off wavelength for HEA-*p*ABA was found to be 265.19 nm, corresponding to the gap energy of 4.68 eV, which is typical of insulating materials. There is no absorption in the entire visible region. This is the most favourable characteristic for a non-linear optical material.

FT-IR analysis indicates salt formation by the presence of bands arising from the asymmetric and symmetric vibrations of the COO^−^ group occurring at 1591 cm^−1^ and 1384 cm^−1^, which do not exist in the spectra of free acid and by the absence of *ν*C = O and *ν*C-OH bands at 1673 cm^−1^ and 1291 cm^−1^, characteristic for –COOH group [49], [50]. At the same time, the band of the deformation vibrations of the N–H bonds is observed at 1540 cm^−1^. A supplementary proof of the salt formation was the appearance of C−O vibrations between 1100 and 1000 cm^−1^, belonging to −CH_2_OH from HEA and a broad band near 2100 cm^−1^, which often appears in amine salt [50] (see Figure S2). The -NH_3_^+^ vibrations appears at 3200–2800 cm^−1^ and δNH_3_^+^ at 2400–2800 cm^−1^. The changes of wavenumbers of the bands of carboxylate group in the case of salt were discussed comparing to the acid. From the spectroscopic data and the elemental analysis it is concluded that *p*ABA reacted with HEA with a 1∶1 ratio.

### Crystal structure analysis of HEA-pABA

**Figure 1:**
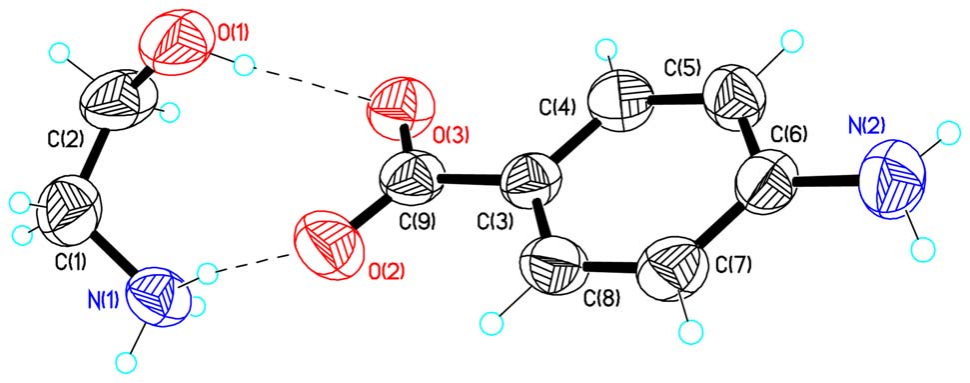
ORTEP drawing for HEA-*p*ABA. The atomic labeling and charge-assisted hydrogen bonds are represented by dashed lines. Thermal ellipsoids are shown with the 50% probability level.

**Figure 2:**
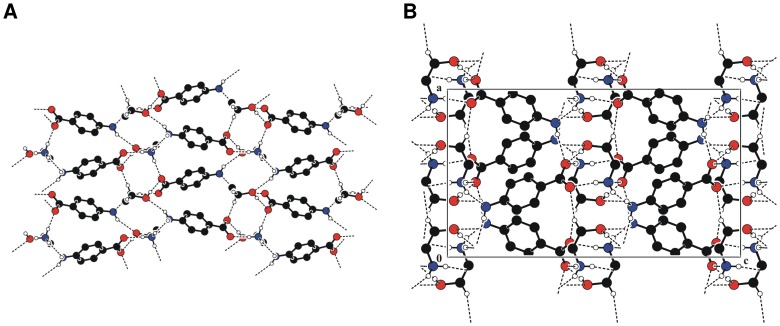
Crystal packing of HEA-*p*ABA. A. Crystal packing of HEA-*p*ABA representing the *2-D* layers parallel to (*100*) plane. B. Fragment of molecular packing in the crystal of HEA-*p*ABA.

The salt unit of HEA-*p*ABA serves as building block for the supramolecular architecture and in the crystal it is self-assembled *via* ionic N-H^…^O and normal O-H^…^O hydrogen bonds (HB) (Figure 1, Table 2). The anion in HEA-*p*ABA forms a practically planar system because the dihedral angle between the least-square plane of the phenyl ring ***A***(C3C4C5C6C7C8) and the least-square plane of the COO^−^ group is equal to 5.8°. The cation adopts the –Syn-Clinal conformation, [30] the N1C1C2O1 torsion angle in HEA-*p*ABA is equal to −68.8°. In the studied compound, the nitrogen atom in amino group has practically a pyramidal configuration because its valence angles are equal to 111.4, 114.9 and 117.2°. It means that the non-bonding electron pair of N2 could be considered as a fourth substituent on a *sp^3^* hybridized nitrogen atom. Thus, the pyramidal geometry of the amino group allows forming three hydrogen bonds in the crystal. Indeed, the building blocks are assembled into *2-D* layers parallel to (*100*) plane (Figure 2A, Table 2) *via* N1^…^O3 (1) HB and two hydrogen bonds with participation of amino group N1^…^N2 (2) and N2^…^O1 (5). These layers are further related along *a* axis by a glide plane perpendicular to [001] with the glide component [0.5 0 0] and a twofold screw axis with direction [100] and screw component [0.5 0 0] due to N2^…^O3 (6) and C2^…^O2 (7) HB respectively (Figure 2B, Table 2). According to the criterion proposed by Spek [30], to establish the presence of π–π stacking interactions between phenyl rings J1 and J2 (Cg(J1)···Cg(J2)<6.0 Å, β<60.0°, where Cg are the respective centroids of the rings and β is the angle between the Cg(J1)Cg(J2) vector and the normal to the J1 ring), there is a stacking interaction between the phenyl rings ***A*** and ***A*** (*−x, 1/2+y, 1/2−z*) within the layers of HEA-*p*ABA. The distance between their centroids is in fact equal to 5.062 Å and β = 16.7°. There is an effect of hydrogen bonding on the geometry of carboxylate groups. While the carboxylate group in HEA-*p*ABA participates as hydrogen-bonding acceptor, the C-O bond lengths vary significantly with the number and type of hydrogen-bonding donors linked to the oxygen atom. In the absence of hydrogen bonding and other electronic perturbations, the C-O bond lengths should be equal because of electron delocalization [23], [24]. Formation of single or multiple hydrogen bonds at one oxygen atom should cause the associated C-O bond to lengthen. In the studied compound, the difference in C9-O2 (1.257(3) Å) and C9-O3 (1.281(3) Å) bond lengths in carboxylate group is equal to 0.024 Å (Table 3). The transfer of proton in HEA-*p*ABA has led to a significant polarization of the anion in comparison with *p*ABA because the calculated dipole moments using HF method [51] for these systems are equal to 13.952 and 3.13 Debye, respectively, and dipole vectors in both molecules lie very close to C3–C9 bond.

### Molecular properties of HEA-pABA and pABA compared to classical auxin molecules

Beside the importance of salt unit of HEA-*p*ABA as building block for supramolecular architecture, we found a new role of this compound and its precursor (*p*ABA) on plant development as potential regulators of plant growth with an auxin-like effect. Auxins are defined by a set of physiological actions: cell division, elongation, formation of lateral and adventitious roots and gravitropism [52]–[54]. A large number of compounds with auxin and anti-auxin activity were identified so far as a result of structure-activity relationship investigations. More than 200 compounds with auxinic action and with different chemical structures have two common features critical for auxin activity: a planar aromatic ring structure and a carboxyl group-containing side chain [55]. The activity of both natural and synthetic auxins depends strongly on side groups binding to the ring structure. Through such substitutions, it is suggested that a positive charged area at specific distance from the carboxylic group is generated. The distance between the negative charge on the carboxyl group and the partial positive charge on the ring has been claimed to be critically important for detectable activity. According to some authors, the optimal distance (OD) appears to be about 5.0–5.5 Å [56], [57]. The ODs in HEA-*p*ABA and *p*ABA were compared with those of classical auxin molecules: indole-3-acetic acid (IAA), 2,4-dichlorophenoxyacetic acid (2,4-D) and 1-naphthaleneacetic acid (1-NAA), where IAA is the most important member of the auxin family and the natural one, the others being synthetic auxin analogs. We performed a search through the Cambridge Structural Database (version 5.34) [58], [59] and found that the crystal structures of these compounds indeed have an OD ranging from 4 to 6 Å. We observed 11, 4, 7 hits for IAA, 1-NAA and 2,4-D where the ODs range from 4.3 to 5.5, 4.3 to 4.6 and 5.1 to 5.3 Å, respectively. The presence of several conformers for IAA, 1-NAA and 2,4-D occurs due to rotation of molecules' moieties on bonds C1(O)-C2 and C2-C3 (see Figure S3). However, only for the conformers of 2,4-D family the ODs satisfy to specific distance between the negative charge on the carboxyl group and the partial positive charge on the ring. Therefore, this result brought us to look for the low energy conformer for IAA and 1-NAA, and then to check the above mentioned criteria for OD values. This was achieved by using the module Conformational Search implemented in HyperChem 6.03 [60]. The method involves the use of the directed scheme as introduced in the Monte Carlo multiple minimum approach [61]. This method seeks to uniformly sample low-energy regions by cycling through all previously accepted conformations (from lowest energy to highest, in order) when selecting each initial structure. In this process, the initial structures from series conformers of IAA and 1-NAA were modified by variation of the torsion angles along the C1–C2 and C2–C3 bonds followed by energy minimization of these angles using the MM+ force field. Then the point charges of atoms were calculated using HF method [51] for found low calculated energy conformers of IAA, 1-NAA and crystal structures of HEA-*p*ABA, *p*ABA [62] and 2,4-D [63]. The results of ODs calculation are summarized in Figure S3. For the studied compounds, the distances between the negative charges on the carboxyl groups and the partial positive charges on the rings ranged from 5.026 to 5.158 Å and the maximal absolute values of point charges which are used for estimation of ODs are observed in HEA-*p*ABA.

### Docking and QM/MM calculations

**Figure 3:**
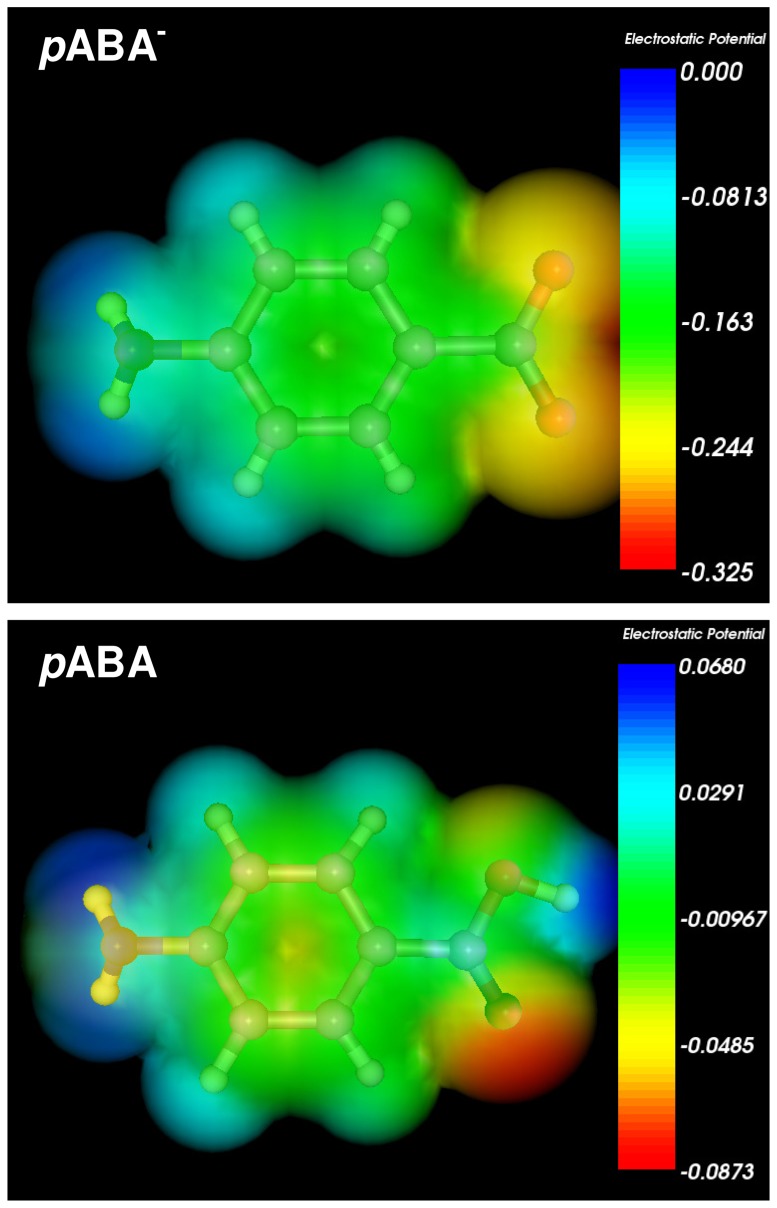
Mapping of the molecular electrostatic potential onto *p*-aminobenzoate anion (*p*ABA^−^) and *p*ABA accessible surface area.

Auxin's receptor, TIR1, belongs to a large family of F-box proteins, which function as part of a protein destruction machinery in the ubiquitin–proteasome system. At the bottom of the TIR1 surface pocket, auxin helps nucleate a hydrophobic core together with TIR1 and its substrate polypeptide [31]. It is known that auxin molecules IAA, 2,4-D, and 1-NAA bind to the TIR1 pocket in a similar manner. Their common carboxyl group interacts with the same positive charged residue at the bottom of the TIR1 pocket [55]. Therefore, in order to estimate the ability of the anionic form of *p*ABA (*p*ABA^−^) and *p*ABA to interact with positive charged residue at the bottom of the TIR1 pocket, we performed the calculation of molecular electrostatic potential map on accessible surface area (Figure 3). In *p*ABA^−^ the region of maximal attractive potential is located in vicinity of both oxygen atoms, while in *p*ABA it is concentrated in vicinity of carbonyl oxygen. Thus, both oxygen atoms in *p*ABA^−^ are able to bind with positive charged residues at the bottom of the TIR1 pocket. In both molecules the attractive potential, which is located in vicinity of the rings, enhances their association with TIR1 pocket.

**Figure 4:**
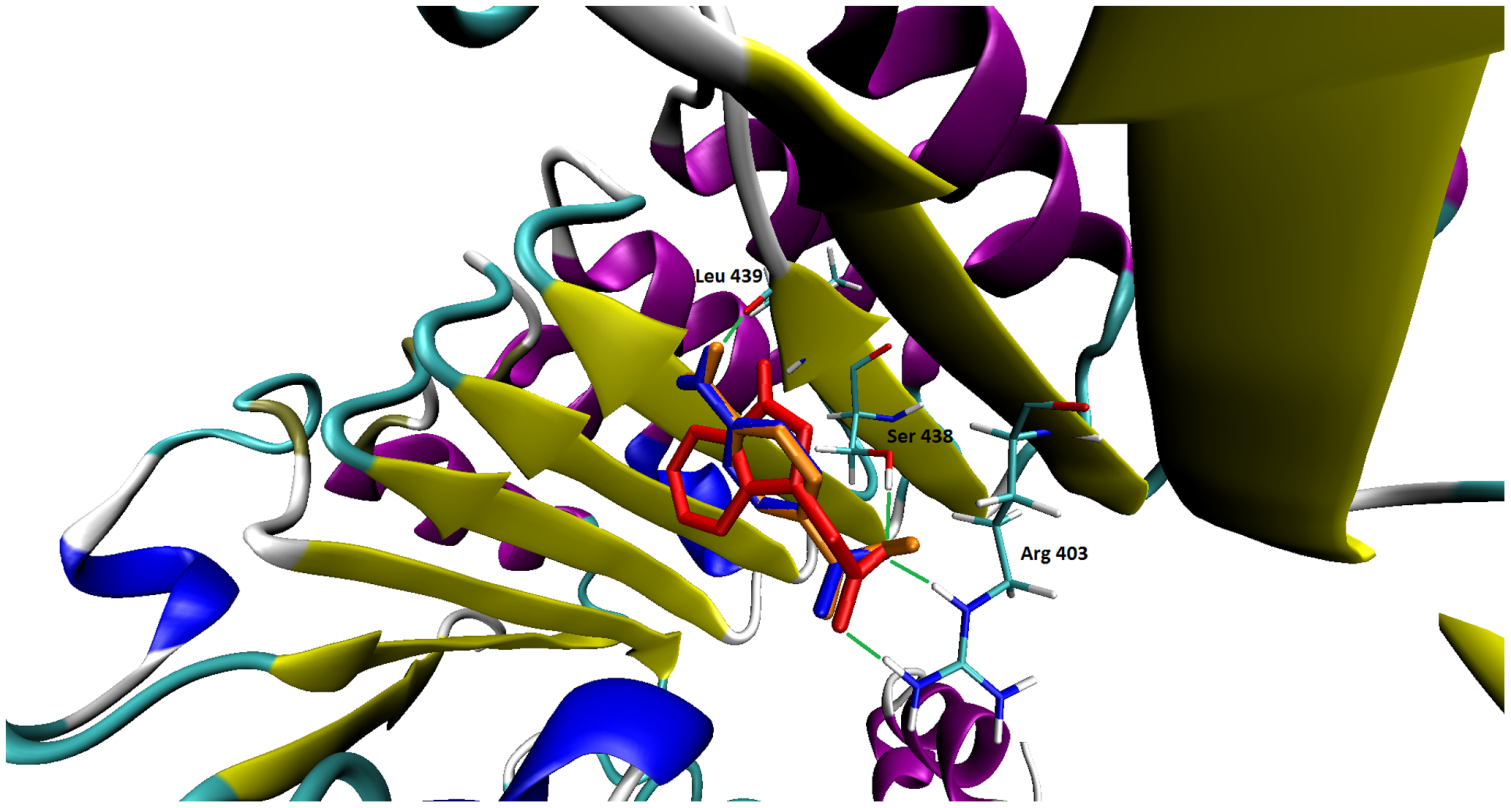
Binding modes of IAA (red), *p*ABA (orange) and *p*ABA^−^ (blue). Hydrogen bonds formed by *p*ABA are drawn as green lines. Only polar hydrogen atoms are shown.

The possible binding modes of *p*ABA to the TIR1 protein were then evaluated by performing docking experiments using the AutoDock 4.2 program. Both the neutral and the anionic form of *p*ABA were docked in the auxin binding site of TIR1 protein as well as auxin itself (IAA), which was redocked to evaluate the performance of the program on this system. The best fit docking poses obtained from the simulation (see Materials and Methods) were then submitted to cluster analysis on a geometrical basis using a 2 Å rmsd threshold. This clustering procedure revealed that in each case only one cluster is present, suggesting a very good convergence of the algorithm. In Figure 4, the docked structure of both neutral and anionic forms of *p*ABA and of auxin IAA are shown. Protonation does not seem to affect binding geometry of *p*ABA. It makes hydrogen bond with Arg403, Ser438 (using the carboxyl function) and with Leu439 backbone oxygen using the amino function. These interactions are the same polar interactions exhibited by IAA, even if in auxin the hydrogen bond of the indolic nitrogen is slightly shorter than the corresponding hydrogen bond in *p*ABA.

Binding energy calculated by AutoDock for the best fit conformations are as follows (in kcal/mol): IAA, −7.91; *p*ABA^−^, −6.19; *p*ABA, −5.97. Again, no significant difference exists between the neutral and the anionic form of *p*ABA, while IAA exhibits a slightly stronger binding to the substrate, probably due to the larger size of the hydrophobic moiety. However, it is to be evidenced the quite small difference in binding energy provided by the three compounds, indicating a similar mode of binding of the proposed ligand with respect to auxin. Molecular dynamics simulations, on the other hand, showed that, while auxin and anionic form of *p*ABA conserve their position and orientation in the binding site compared to the docked structures, the neutral *p*ABA is expelled from the binding site during the simulation. Accordingly, the corresponding Root Mean Square Deviations (RMSD) of the three ligands during simulation with respect to the MD starting structure show different trends (see Figure S4). While auxin IAA and anionic form of *p*ABA display small and well equilibrated RMSD values, for neutral *p*ABA the RMSD increase progressively up to more than 8 Å after 10000 ps, indicating a loss of the starting structure as a consequence of its expulsion from the binding site. The MD results therefore suggest that the salt bridge interaction between Arg403 and the charged carboxyl group on auxin or *p*ABA^−^ is crucial for the interaction of ligands with TIR1 protein.

Geometry optimizations at the hybrid QM/MM level were performed on the final MD structures of auxin and *p*ABA^−^ complex. The QM/MM optimized structure of the TIR1-auxin complex revealed no substantial modification with respect to the docked structures. The HB interactions between Arg403 and Ser438 and auxin carboxyl group are preserved, as well as the HB between the indolic nitrogen and the backbone carbonyl of Leu439 (see Figure S4). In the QM/MM optimised structure of the TIR1-*p*ABA^−^ complex, interaction of the carboxyl group with Arg403 and Ser438 is preserved, while no HB is formed between the amino group of *p*ABA and Leu439 (see Figure S5). The binding energies evaluated at the QM/MM level are −117.0 kcal/mol and −85.6 kcal/mol for IAA and *p*ABA^−^, respectively, in agreement with the observed differences in the network of interactions between the protein and the two ligands.

It is worth noting that the larger magnitude of binding energies with respect to the values obtained by docking calculations should be ascribed to the fact that QM/MM calculations have been performed *in vacuo*, owing to the high complexity of the investigated systems. Within such approximation, the electrostatic interaction necessarily results to be overestimated with respect to the more realistic values obtained by docking experiments, where the solvent effect is implicitly taken into account by the score function used. In spite of such approximation, QM/MM calculations appear to confirm the similar interaction of anionic form of *p*ABA with respect to auxin IAA. It is to be noted that very similar results have been recently obtained on the binding mechanisms of neutral and charged phenolic compounds with the human transthyretin, hTTR, using a similar approach combining molecular docking with QM/MM optimizations [64]. In agreement with their conclusions, ionization effects should not be neglected when modeling the binding modes of ligands with proteins.

### HEA-pABA and pABA activity on root growth

**Figure 5:**
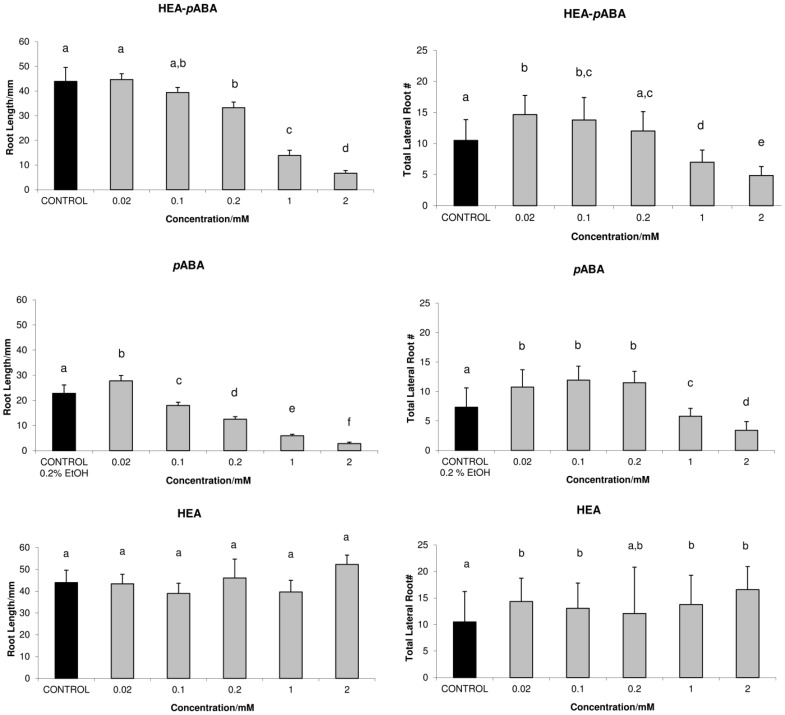
Root length and number of lateral roots after 10 days of treated *A. thaliana* seedlings. HEA-*p*ABA, *p*ABA and HEA treatments at different concentrations in comparison with control are shown. Control corresponding to *p*ABA was noted CONTROL 0.2% EtOH and for HEA-*p*ABA and HEA, CONTROL. Values with different letter annotation are significantly different (P<0.05). Data are means ± SE of 5 replicates.

Phytotoxicity tests, especially the seed germination and root elongation tests, have more advantages over those toxicity tests using animals and algae, such as sensitivity, simplicity and low cost [65]–[67]. Phytotoxicity of HEA-*p*ABA, *p*ABA and HEA were determined at five different concentrations. Seeds of the model plant *Arabidopsis thaliana* have been used for germination, early seedling growth and gravity response tests. Figure 5 shows wild-type (WT) *A. thaliana* root growth on a medium supplemented with 0.02, 0.1, 0.2, 1 and 2 mM of HEA-*p*ABA, *p*ABA and HEA as compared to controls after 5 and 10 days (see also Figure S6). HEA-*p*ABA and *p*ABA showed a clear difference in primary root growth, and lateral root development after prolonged growth (10 days) (Figure 5). Moreover, the root growth difference was already apparent after 5 days, indicating that the action of tested compounds occurs throughout the period of elongation growth (see Figure S6). After 10 days, HEA treated seedlings exhibited a comparable effect to control for primary root length and an increased number of lateral roots at all concentrations tested. At the lowest concentration (0.02 mM), *p*ABA treatment showed an increased number of lateral roots and primary root length, a common anti-auxin feature (Figure 5). At this concentration, HEA-*p*ABA displays a comparable effect to control, regarding the primary root length and an increased number of lateral roots. At 0.1 and 0.2 mM, both HEA-*p*ABA and *p*ABA promote lateral roots, while they inhibit primary root length (Figure 5). The phenotypes like short root length, increased lateral root number are phenotypes similar to those observed in *A. thaliana* treated with auxins [68]–[70]. At high concentration (2 mM), primary root length is inhibited but also displayed a different growth behavior with respect to control seedlings. Preliminary tests aimed to compare HEA-*p*ABA, *p*ABA and IAA effects indicate that the latter exerts a 1000-fold higher root length inhibition (data not shown). The agravitropic effect of root growth is specific for *p*ABA and HEA-*p*ABA, never having been reported for an auxinic compound. Therefore, we argue that HEA-*p*ABA and *p*ABA might potentially act as auxin-like regulators of root development in *A. thaliana* (0.1 and 0.2 mM) and display an agravitropic root response at high concentration (2 mM) (see Figure S7).

## Conclusions

This study presents the synthesis and the unique structural and biological properties of HEA-*p*ABA. In the crystal, the salt unit of HEA-*p*ABA serves as building block of the supramolecular architecture and in the crystal it is self-assembled *via* ionic N-H^…^O and normal O-H^…^O hydrogen bonds. The pyramidal geometry of *p*ABA amino group allows to form three hydrogen bonds in the crystal. The transfer of proton in HEA-*p*ABA has led to significant polarization of anion in comparison with *p*ABA and the absolute values of point charges, which are used for estimation of ODs, are higher in HEA-*p*ABA. For both these compounds, ODs are equal to 5.045 and 5.026 Å respectively. Both oxygen atoms in anionic form of *p*ABA are able to bind with positive charged residues at the bottom of the TIR1 pocket. In *p*ABA^−^ and *p*ABA the molecular electrostatic attractive potential, which is located in vicinity of the rings, enhances their association with TIR1 pocket. Docking experiments on complexes of *p*ABA (in both neutral and anionic form) and IAA with TIR1 provide a similar binding mode between the two ligands. MD and QM/MM calculations confirm such result as far as *p*ABA^−^ is concerned. Phytotoxicity test indicates that compound HEA-*p*ABA and *p*ABA act as auxin-like regulators of root development in *A. thaliana* (0.1 and 0.2 mM) and display an agravitropic root response at higher concentration (2 mM). This study suggests for these compounds a potential plant growth regulatory activity of the auxin type. Between the two ionic species, anionic from acid and cationic from HEA, the anionic species presented an increased phytotoxic effect. However, up to now, the plant growth regulatory properties of HEA-*p*ABA and *p*ABA have not been found in literature, which is needed in order to develop its new application fields and carry out relevant theoretical research.

## Supporting Information

Figure S1 **UV absorption spectra of HEA-** ***p*** **ABA and** ***p*** **ABA in 0.1 M NaOH solution.**
(PDF)

Figure S2 **FT-IR spectra of HEA-** ***p*** **ABA (black) and** ***p*** **ABA (red).**
(PDF)

Figure S3 **Optimal distance calculation for HEA-** ***p*** **ABA,** ***p*** **ABA and classical auxin molecules (IAA, 1-NAA, 2,4-D).**
(PDF)

Figure S4 **(A) Binding mode of IAA with TIR1.** Hydrogen bonds are drawn as green lines. **(B). Root Mean Square Deviation of the ligand with respect to the MD starting structure.**
(PDF)

Figure S5 **Binding mode of** ***p*** **ABA^−^ with TIR1.** Hydrogen bonds are drawn as green lines.
(PDF)

Figure S6 **Root length (after 5 and 10 days) of treated** ***A. thaliana*** **seedlings.** HEA-*p*ABA, *p*ABA and HEA treatments at different concentrations in comparison with control. Values with different letter annotation are significantly different (P<0.05). Data are means ± SE of 5 replicates.
(PDF)

Figure S7 **Effects of HEA-** ***p*** **ABA,** ***p*** **ABA and HEA on root development of** ***A. thaliana*** **(Columbia 0) seedlings.** The 10 day old *A. thaliana* seedlings were germinated on vertical agar plates. Scale bars, 0.5 cm.
(PDF)
