# Supplementary figures and images for: Synthesis, Crystal Structure and Biological Activity of 2-Hydroxyethylammonium Salt of p-Aminobenzoic Acid
Source: PLoS One. 2014 Jul 23;9(7):e101892. doi: 10.1371/journal.pone.0101892 (PMC4108362; doi:10.1371/journal.pone.0101892)

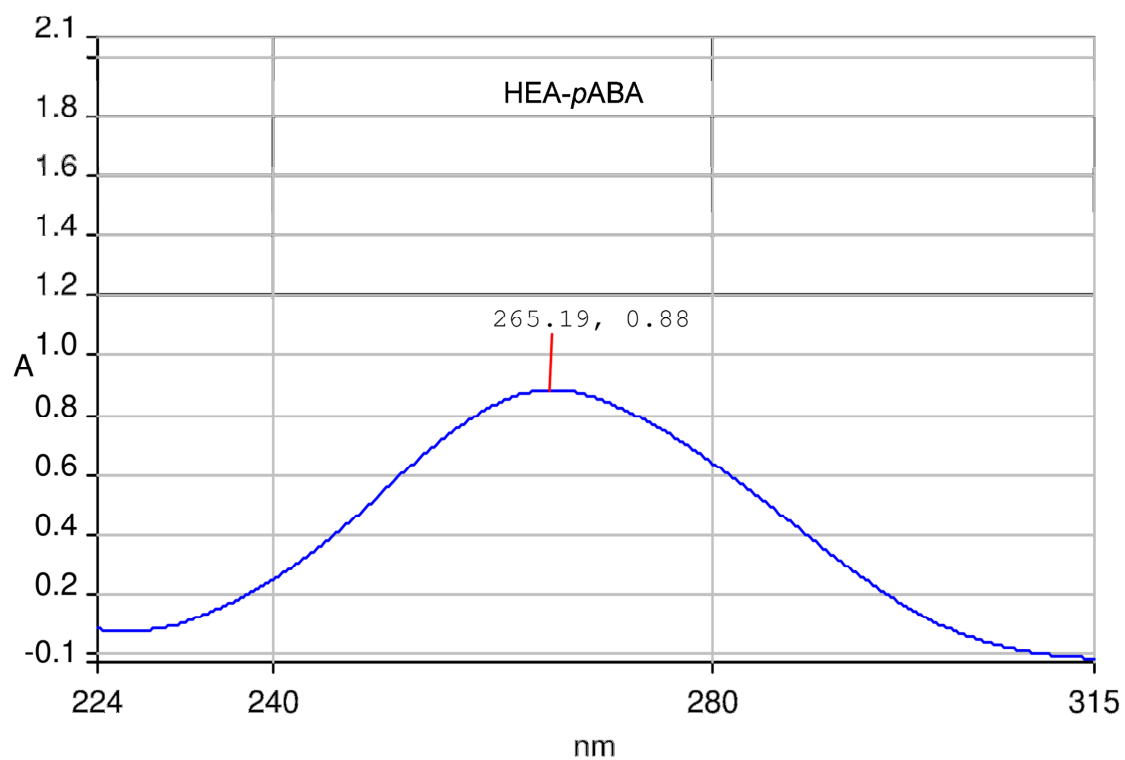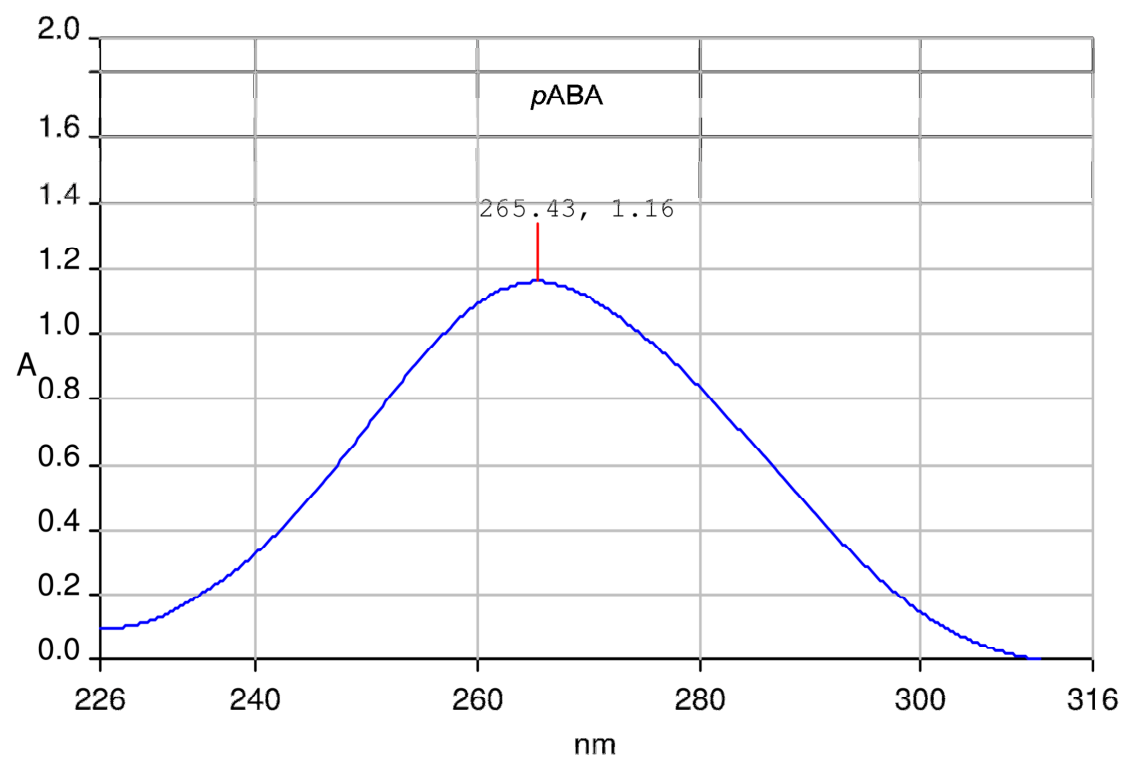

**Figure S1.** UV absorption spectra of HEA-pABA and pABA in 0.1 M NaOH solution.

Supplement: Figure S1 — UV absorption spectra of HEA- p ABA and p ABA in 0.1 M NaOH solution. (PDF) [file pone.0101892.s001.pdf]

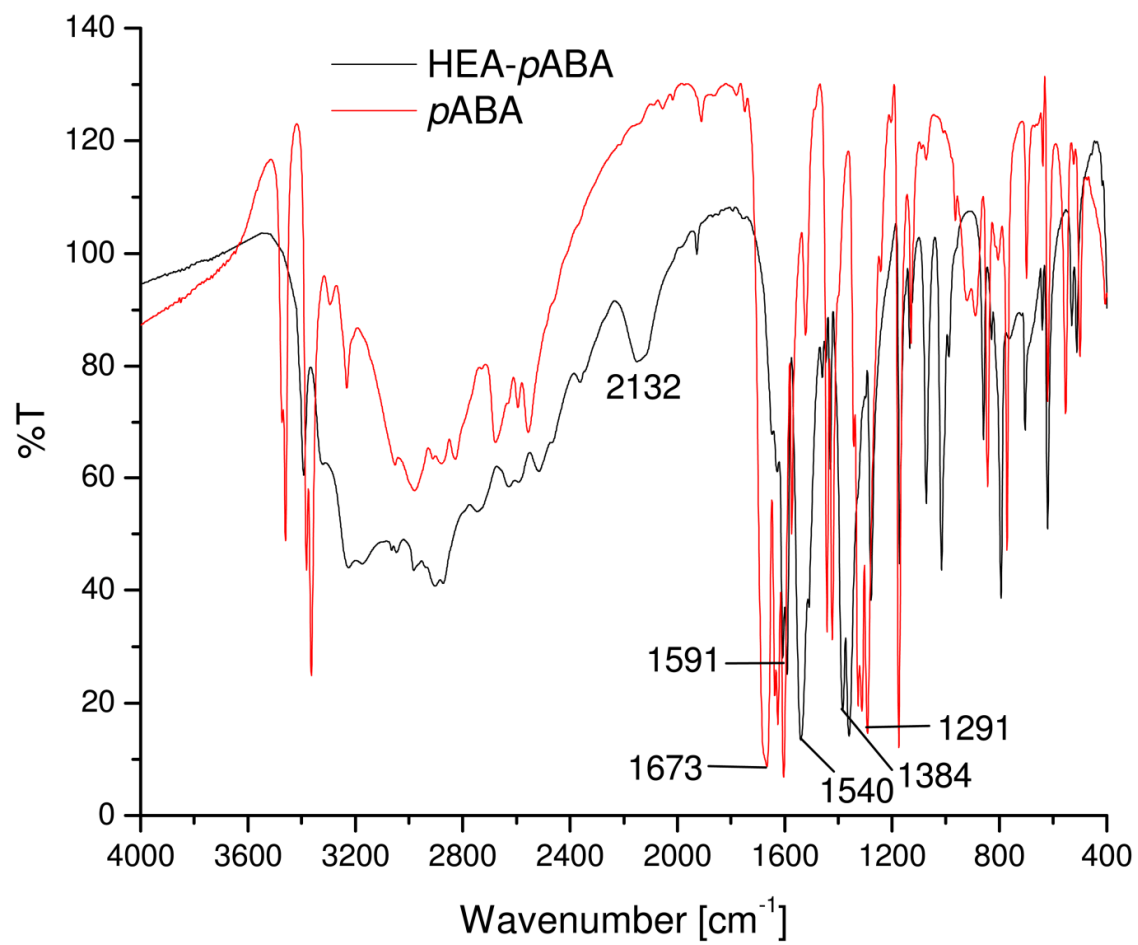

**Figure S2. FT-IR spectra of HEA-*p*ABA (black) and *p*ABA (red).**

Supplement: Figure S2 — FT-IR spectra of HEA- p ABA (black) and p ABA (red). (PDF) [file pone.0101892.s002.pdf]

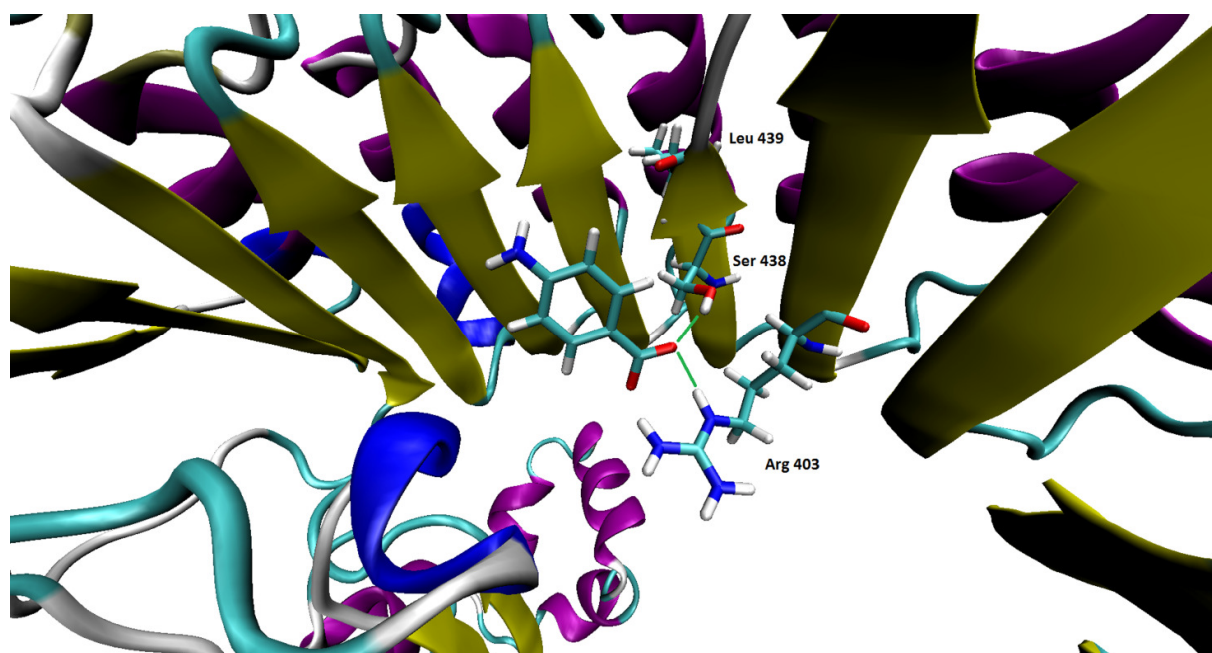

**Figure S5. Binding mode of *pABA*<sup>-</sup> with TIR1.** Hydrogen bonds are drawn as green lines.

Supplement: Figure S5 — Binding mode of p ABA− with TIR1. Hydrogen bonds are drawn as green lines. (PDF) [file pone.0101892.s005.pdf]
