# Supplementary material for: Synthesis, Crystal Structure and Biological Activity of 2-Hydroxyethylammonium Salt of p-Aminobenzoic Acid
Source: PLoS One. 2014 Jul 23;9(7):e101892. doi: 10.1371/journal.pone.0101892 (PMC4108362; doi:10.1371/journal.pone.0101892)
Supplement: Figure S3 — Optimal distance calculation for HEA- p ABA, p ABA and classical auxin molecules (IAA, 1-NAA, 2,4-D). (PDF) [file pone.0101892.s003.pdf]

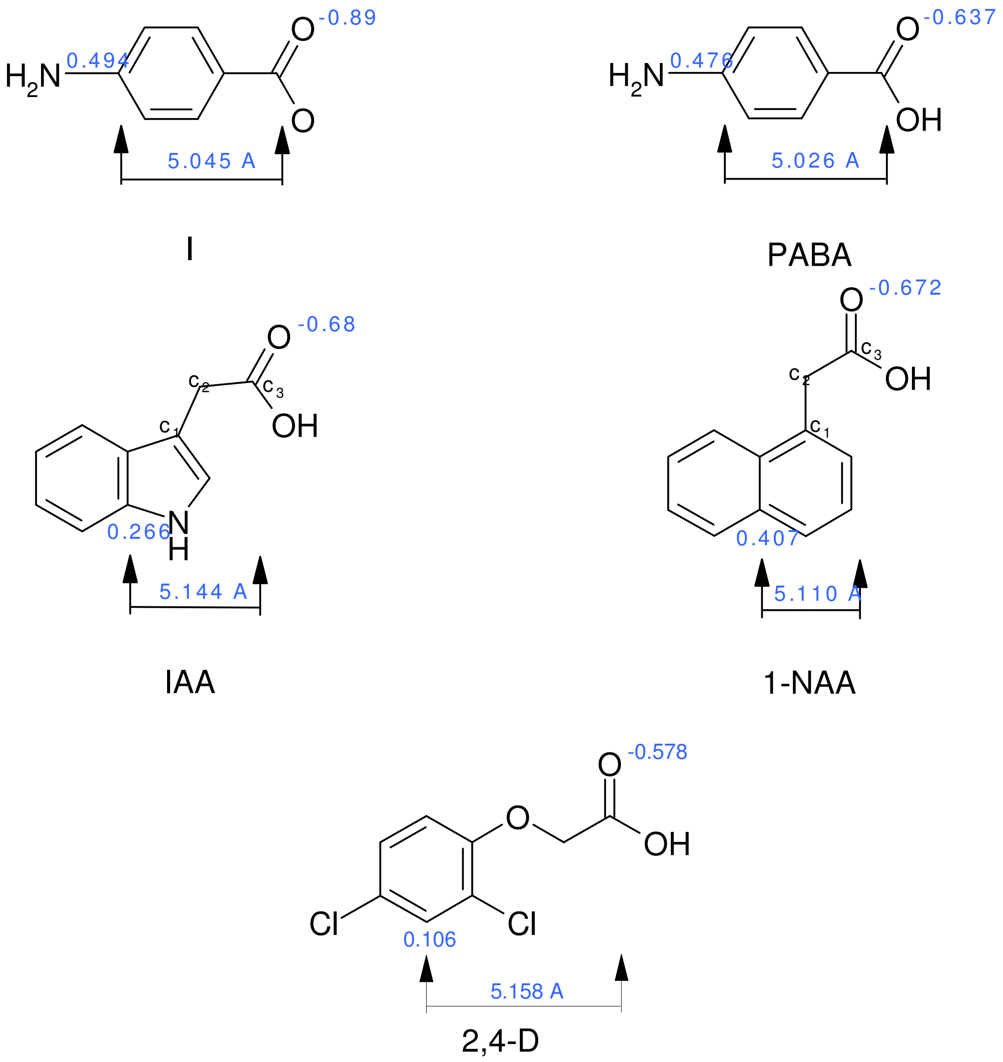

Figure S3. Optimal distance calculation for HEA-*p*ABA, *p*ABA and classical auxin molecules (IAA, 1-NAA, 2,4-D).
