# Supplementary material for: Synthesis, Crystal Structure and Biological Activity of 2-Hydroxyethylammonium Salt of p-Aminobenzoic Acid
Source: PLoS One. 2014 Jul 23;9(7):e101892. doi: 10.1371/journal.pone.0101892 (PMC4108362; doi:10.1371/journal.pone.0101892)
Supplement: Figure S4 — (A) Binding mode of IAA with TIR1. Hydrogen bonds are drawn as green lines. (B). Root Mean Square Deviation of the ligand with respect to the MD starting structure. (PDF) [file pone.0101892.s004.pdf]

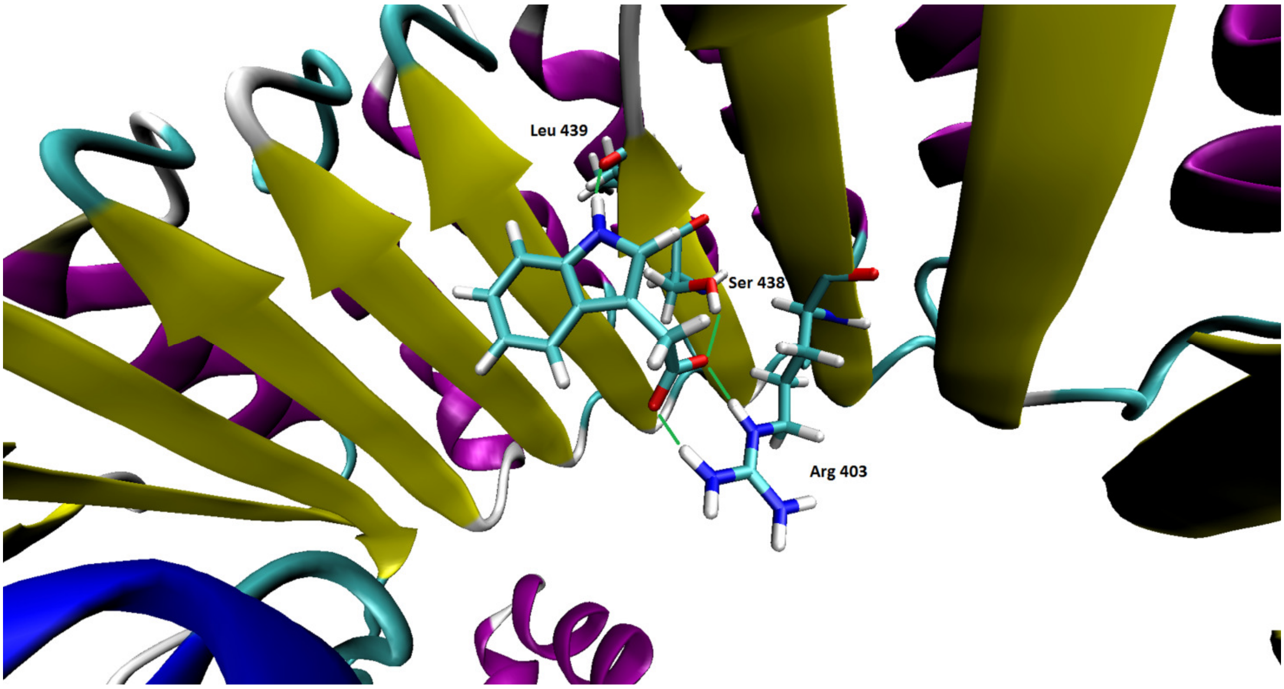

Figure S4a. Binding mode of IAA with TIR1. Hydrogen bonds are drawn as green lines.

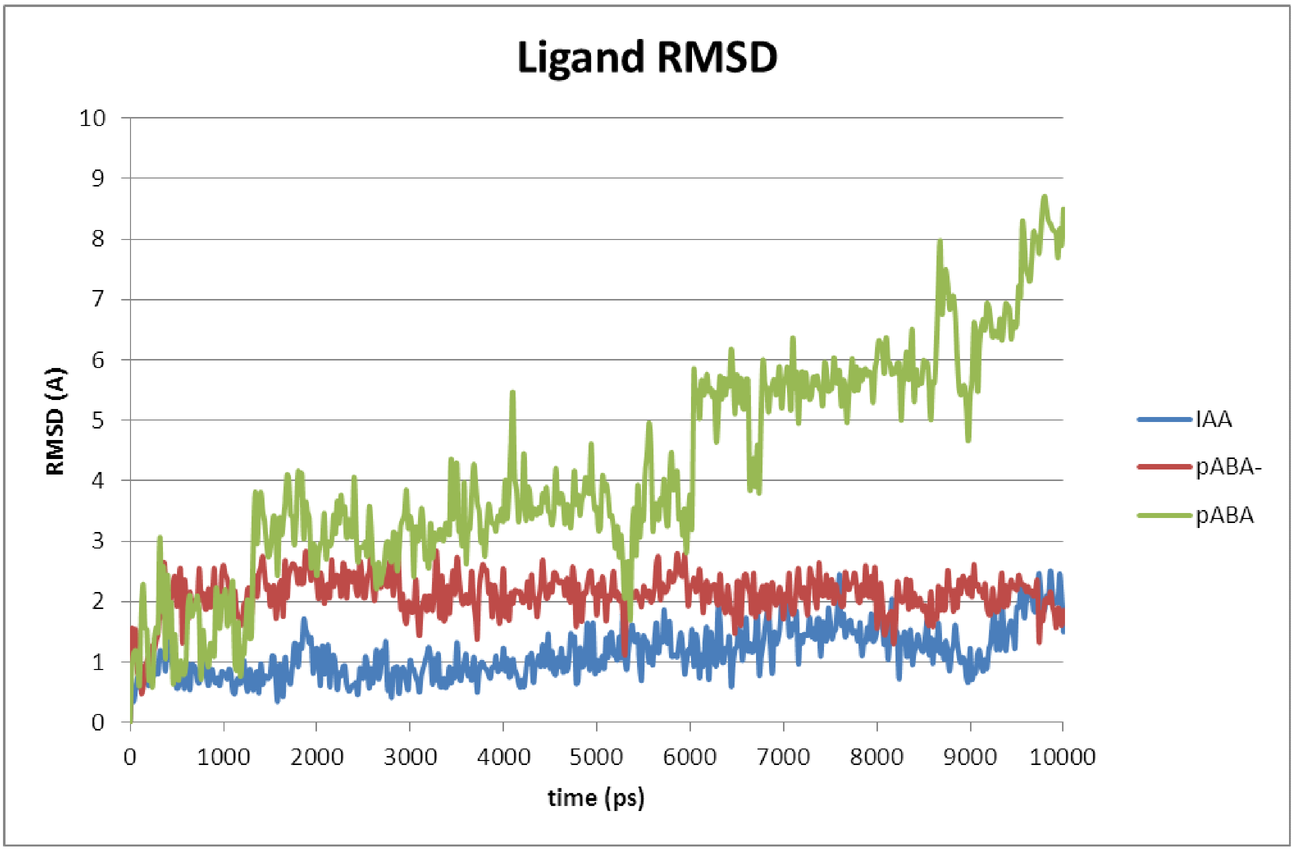

Figure S4b. Root Mean Square Deviation of the ligand with respect to the MD starting.
