# Supplementary material for: Synthesis, Crystal Structure and Biological Activity of 2-Hydroxyethylammonium Salt of p-Aminobenzoic Acid
Source: PLoS One. 2014 Jul 23;9(7):e101892. doi: 10.1371/journal.pone.0101892 (PMC4108362; doi:10.1371/journal.pone.0101892)
Supplement: Figure S6 — Root length (after 5 and 10 days) of treated A. thaliana seedlings. HEA-pABA, pABA and HEA treatments at different concentrations in comparison with control. Values with different letter annotation are significantly different (P<0.05). Data are means ± SE of 5 replicates. (PDF) [file pone.0101892.s006.pdf]

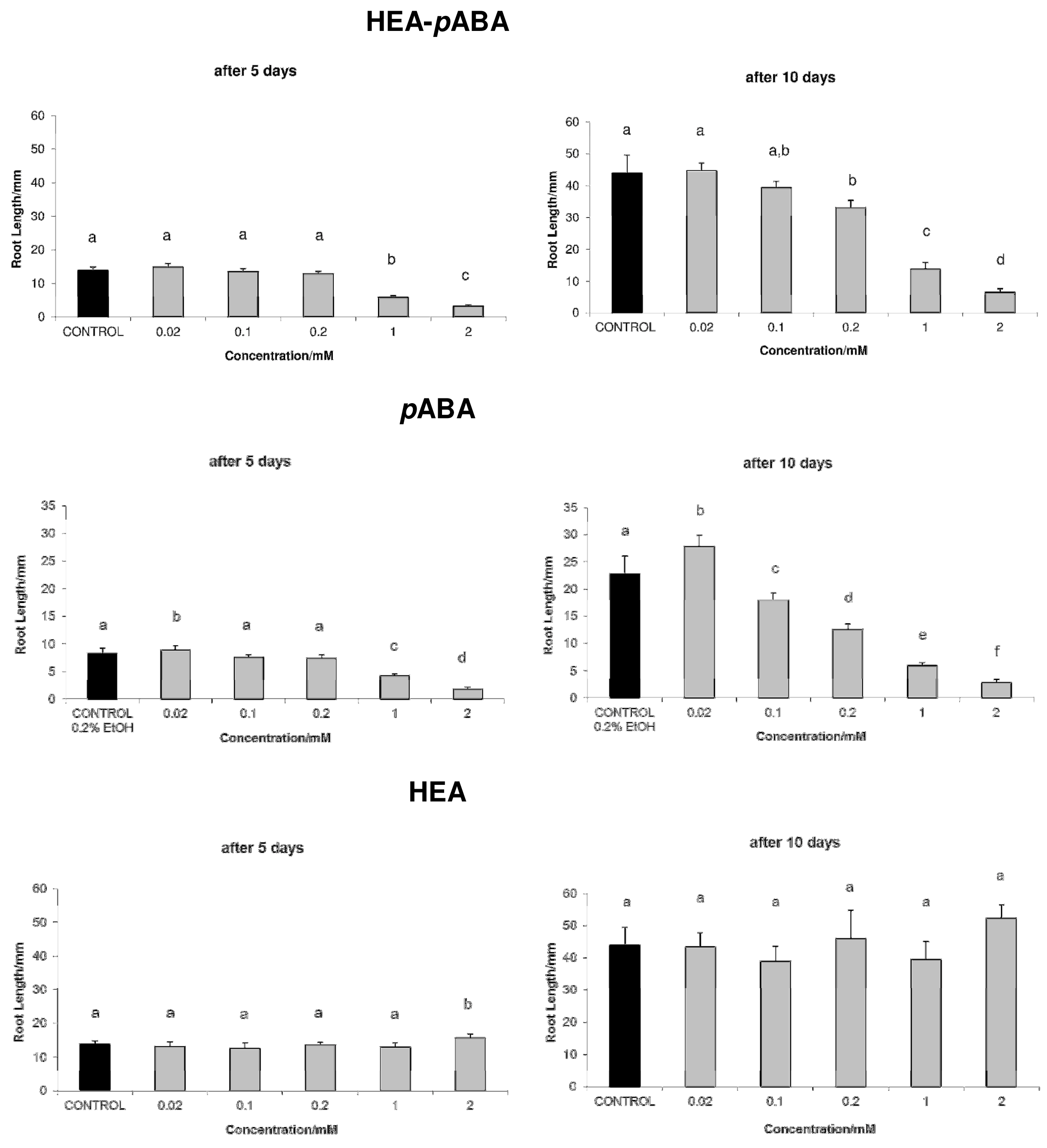

Figure S6. Root length (after 5 and 10 days) of treated *A. thaliana* seedlings. HEA-*p*ABA, *p*ABA and HEA treatments at different concentrations in comparison with control. Values with different letter annotation are significantly different ($P<0.05$). Data are means ± SE of 5 replicates.
