# Supplementary material for: Synthesis, Crystal Structure and Biological Activity of 2-Hydroxyethylammonium Salt of p-Aminobenzoic Acid
Source: PLoS One. 2014 Jul 23;9(7):e101892. doi: 10.1371/journal.pone.0101892 (PMC4108362; doi:10.1371/journal.pone.0101892)
Supplement: Figure S7 — Effects of HEA- p ABA, p ABA and HEA on root development of A. thaliana (Columbia 0) seedlings. The 10 day old A. thaliana seedlings were germinated on vertical agar plates. Scale bars, 0.5 cm. (PDF) [file pone.0101892.s007.pdf]

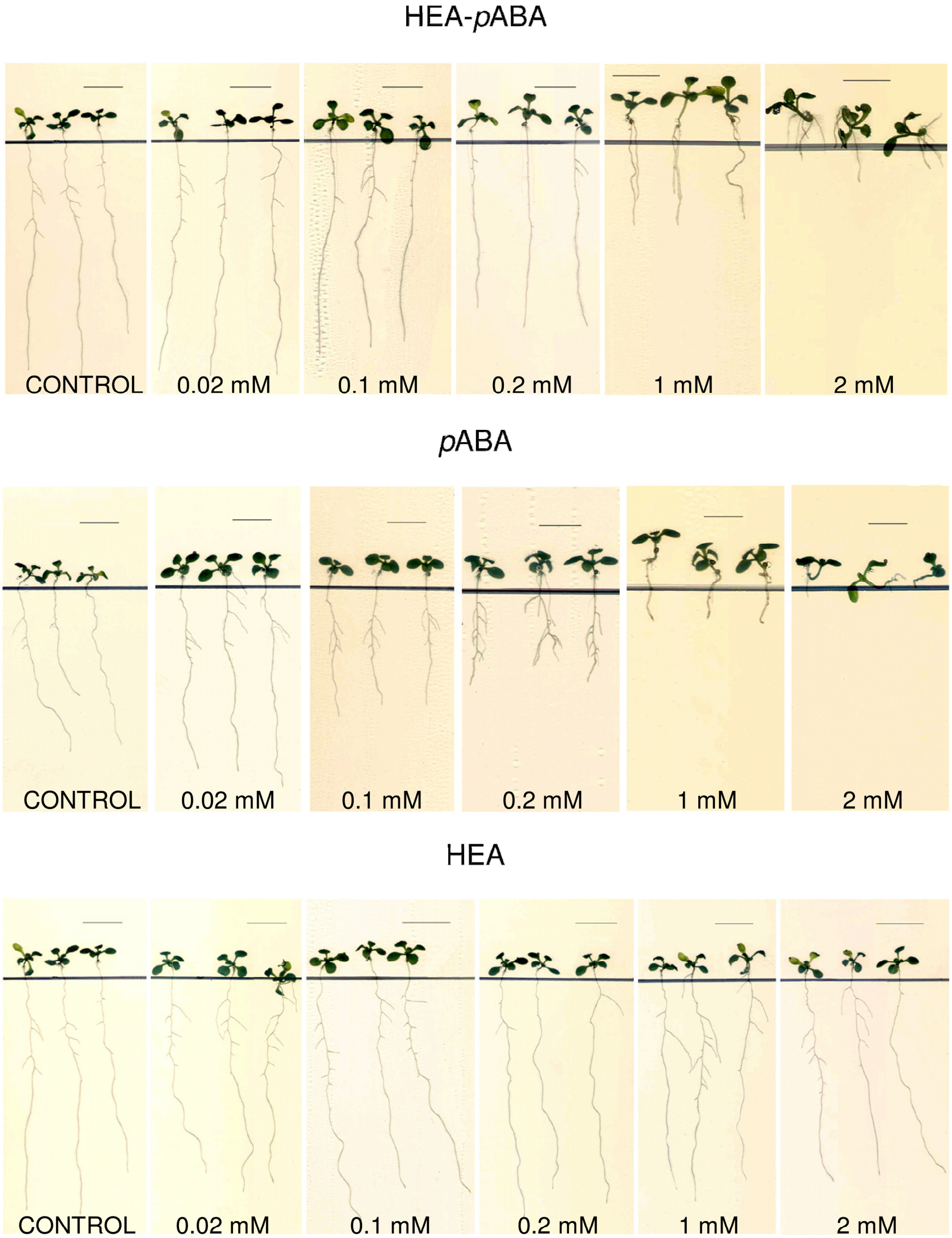

Figure S7. Effects of HEA-*p*ABA, *p*ABA and HEA on root development of *A. thaliana* (Columbia 0) seedlings. The 10 day old *A. thaliana* seedlings were germinated on vertical agar plates. Scale bars, 0.5 cm.
